# Survey response over 15 years of follow-up in the Millennium Cohort Study

**DOI:** 10.1186/s12874-023-02018-z

**Published:** 2023-09-09

**Authors:** Claire A. Kolaja, Jennifer N. Belding, Satbir K. Boparai, Sheila F. Castañeda, Toni Rose Geronimo-Hara, Teresa M. Powell, Xin M. Tu, Jennifer L. Walstrom, Beverly D. Sheppard, Rudolph P. Rull, for the Millennium Cohort Study Team

**Affiliations:** 1 Deployment Health Research Department, Naval Health Research Center, San Diego, CA USA; 2 Leidos, Inc, San Diego, CA USA; 3 Army Resilience Directorate, Headquarters United States Department of the Army, Deputy Chief of Staff G-1, Arlington, VA USA; 4 Clinical and Translational Research Institute, University of California San Diego, San Diego, CA USA

**Keywords:** Follow-up surveys, Cohort study, Military, Longitudinal, Response rates

## Abstract

**Background:**

Patterns of survey response and the characteristics associated with response over time in longitudinal studies are important to discern for the development of tailored retention efforts aimed at minimizing response bias. The Millennium Cohort Study, the largest and longest running cohort study of military personnel and veterans, is designed to examine the long-term health effects of military service and experiences and thus relies on continued participant survey responses over time. Here, we describe the response rates for follow-up survey data collected over 15 years and identify characteristics associated with follow-up survey response and mode of response (paper vs. web).

**Method:**

Patterns of follow-up survey response and response mode (web, paper, none) were examined among eligible participants (*n=*198,833), who were initially recruited in four panels from 2001 to 2013 in the Millennium Cohort Study, for a follow-up period of 3–15 years (2004–2016). Military and sociodemographic factors (i.e., enrollment panel, sex, birth year, race and ethnicity, educational attainment, marital status, service component, service branch, pay grade, military occupation, length of service, and time deployed), life experiences and health-related factors (i.e., military deployment/combat experience, life stressors, mental health, physical health, and unhealthy behaviors) were used to examine follow-up response and survey mode over time in multivariable generalized estimating equation models.

**Results:**

Overall, an average response rate of 60% was observed across all follow-up waves. Factors associated with follow-up survey response over time included increased educational attainment, married status, female sex, older age, military deployment (regardless of combat experience), and higher number of life stressors, mental health issues, and physical health diagnoses.

**Conclusion:**

Despite the challenges associated with collecting multiple waves of follow-up survey data from members of the U.S. military during and after service, the Millennium Cohort Study has maintained a relatively robust response rate over time. The incorporation of tailored messages and outreach to those groups least likely to respond over time may improve retention and thereby increase the representativeness and generalizability of collected survey data.

**Supplementary Information:**

The online version contains supplementary material available at 10.1186/s12874-023-02018-z.

## Background

Prospective cohort studies follow a group of participants and collect data on them over a period of time to answer important causal research questions related to emergence of disease in relation to known and unknown exposures [1]. In the early 2000s, cohort studies began to observe a precipitous decline in responses to follow-up surveys [2–6]. In addition, several characteristics were consistently associated with attrition, or loss to follow-up, in cohort studies, including male sex, younger age, single marital status, and lower socioeconomic status [7–9]. Differential response rates among participants to follow-up surveys can negatively affect the validity and generalizability of study findings [10, 11].

Prior to the wide availability of self-administered internet surveys, researchers often relied on paper-and-pencil surveys that were mailed back to the study operations center or administered in person. Reliance on paper-based surveys can be expensive and cost prohibitive in terms of materials, postage, and software and labor for data entry and cleaning. With the widespread increase in internet usage in the United States (from 55 to 93% between 2000 and 2021 [12]) survey data can now be collected remotely, which is often more cost effective, less labor intensive to process the data, and carries additional advantages including the ability to program complex skip logic [13–15]. However, due to issues such as disparities in internet access, certain groups, such as those from racial or ethnic minority groups or who are older, of lower socioeconomic status, or reside in rural communities, may be underrepresented in studies that employ online surveys as their sole mode of data collection [12]. To mitigate these concerns and boost response rates, some studies employ a multimodal survey design where participants can complete either a paper or a web-based survey [16]. However, more research is needed to understand the characteristics that may be associated with mode of survey response in longitudinal cohort studies over time, particularly those focused on military personnel and other hard-to-reach populations.

**Fig. 1:**
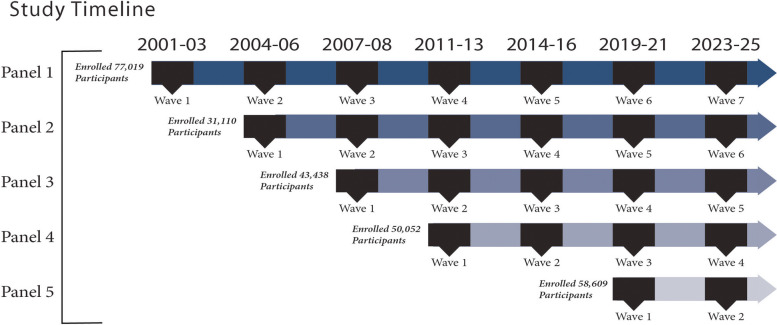
Depiction of the multiple panel design used in the Millennium Cohort Study

The Millennium Cohort Study (hereafter the Study), the largest and longest running cohort study of service members and veterans, was initiated in 2001 with the objective of studying the long-term health impacts of military deployments. Detailed summaries of Study methodology are available elsewhere [17–19]. Briefly, the Study employs a multi-panel, multi-wave design in which different cohorts (called panels) of service members are enrolled and invited to complete follow-up surveys in cycles called waves via a website or paper mode approximately every 3–5 years (Fig. 1) [20].

Participants from the first four panels (*N=*201,619) were invited to complete at least one follow-up survey as of 2016. Examinations of the representativeness and response patterns of the Study have been previously reported [18, 20–22], but they were limited to participants from the first enrollment panel. While findings suggested that Panel 1 was representative of the general military at baseline [18, 21], there were observed differences in response rates at the first follow-up survey (called Wave 2, which was conducted between 2004 and 2006 for Panel 1). However, additional longitudinal patterns of response rates over multiple follow-up waves were not examined beyond Wave 2 for Panel 1. The aims of the current analysis are to (1) report the overall and by mode (paper vs. web) response rates between the second and fifth waves spanning up to 15 years of follow-up, and (2) identify participant characteristics associated with response and mode of response across two to five waves of survey collection.

## Methods

### Participants

Across the five panels, 260,228 participants enrolled in the Study between 2001 and 2021 (enrolled in 2001–2003, 2004–2006, 2007–2008, 2011–2013, and 2020–2021). While Panel 1 was invited from a random sample of service members from active duty and Reserve/National Guard rosters in October 2000, subsequent panels randomly sampled more recently accessed service members (i.e., with 1–5 years of military service) and oversampled certain subgroups (e.g., women, Marine Corps personnel) to enable sufficient sample size for between-group comparisons. At the start of each follow-up wave, participants were excluded if they withdrew from the study or were deceased. Panel 1 participants were eligible for five follow-up waves, but each subsequent panel was eligible for fewer follow-up waves due to the staggered multi-panel and multi-wave study design (Fig. 1).

### Methods to increase follow-up survey response

Historically, the Study employed multiple strategies to increase follow-up survey response such as targeting outreach (e.g., contacts intended for specific service branches or veterans), updating contact information from multiple sources, and using incentives. Some techniques, such as postcard and email reminders to complete the survey, were consistently utilized over time, but the frequency and timing of those techniques varied by survey cycle (Table 1). Reminder emails notified participants to start or complete the web survey and postcards were sent to nonresponders. During the 2007–2008 and 2011–2013 survey cycles, the Study used reminder voice messages and during the 2014–2016 survey cycle, one primer email was sent before the survey cycle began. The Study also sent out annual commemorative emails and postcards for Memorial Day and Veterans Day that served as reminders for participants to complete the survey if the holiday fell within a survey cycle. Endorsement letters from Department of Defense leadership were sent to participants during the 2011–2013 and 2014–2016 surveys. Type of incentives and timing of distribution (either before or after survey completion) changed over time; an experiment conducted during the 2014–2016 cycle found that monetary pre-incentives produced a modest increase in follow-up survey response [23]. Finally, during the 2014–2016 survey cycle a short (2-page) paper survey was created and mailed to nonresponders 18 and 20 months into the cycle to increase response.

**Table 1:** Millennium Cohort Study follow-up survey methods between 2004–2006 and 2014–2016

	Follow-up Survey Cycle Years			
	2004—2006	2007—2008	2011—2013	2014—2016
Months cycle was open	20	19	24	23
Primer email	No	No	No	Yes
Number of reminder emails^a^	10	23	22	21
Number of reminder postcards^b^	4	4	3	3
Number of reminder automated phone message	0	3	1	0
Commemorative and reminder emails and postcards (Memorial Day and Veterans Day)	Yes	Yes	Yes	Yes
Endorsement letter	No	No	Yes^c^	Yes^c^
Pre-incentive letter	No	No	No	1
Newsletter	No	No	No	2
Desktop calendar	No	No	No	1
Web survey completion post-incentive	Choice between hat, t-shirt, or phone card	Choice between hat, t-shirt, or phone card	Choice between $5 gift card to Starbucks, Walmart, Amazon, or Subway, hat, coin, or none;	Choice between $5 gift card to Starbucks, Walmart, Amazon or Subway, hat, or none
Pre-incentive	None	Gift card sent to late paper survey responders at end of cycle	$5 for incomplete surveys at end of cycle	Randomly assigned experiment: magnet; $2 bill; gift card; lottery entry for an iPad; or letter/no pre-incentive
Number of paper questionnaires	4	5	5	4
Month of survey cycle the paper questionnaires were mailed	2, 4, 6, 12	1, 3, 6, 11, 15	3, 5, 9, 17, 19	3, 7, 12, 15
Number of short paper (2 page) questionnaires mailed	0	0	0	2
Month of survey cycle the short paper (2 page) questionnaires were mailed	-	-	-	18, 20

^a^Those who did not start the web survey were reminded of the importance of the study and staying involved. Those who started and did not complete the web survey were reminded to complete the survey

^b^Sent to nonresponders

^c^2011–13 from Chairman of the Joint Chiefs of Staff; 2014–16 from Surgeon General of the Navy, Surgeon General of the Army, and Chairman of the Joint Chiefs of Staff

### Measures

#### Military and demographic covariates

Demographic and military service factors such as birth year, sex, race and ethnicity, pay grade, service branch, military occupation, length of service (LOS), and service component were obtained from administrative records maintained by the Defense Manpower Data Center (DMDC). Military factors were measured concurrent with the follow-up response since they could be obtained from administrative records and did not rely on participant response. All other self-reported factors listed in the measures section were measured as ever occurring before each follow-up wave. Deployment dates were obtained from the Contingency Tracking System (CTS) and were used to calculate the total years deployed before each follow-up survey. Educational attainment and marital status were self-reported on the survey and backfilled with DMDC data if missing.

#### Stressful life events

Combat deployment experience was identified using a combination of administrative CTS records with a 5-item combat experience scale on the survey with responses categorized into one of four categories (i.e., deployed with combat, deployed without combat, deployed with unknown combat, not deployed) consistent with prior research [24]. Life stressors were assessed from five modified items (i.e., divorce, financial issues, sexual assault, sexual harassment, and physical assault) from the Holmes and Rahe Social Readjustment Rating Scale, and participants were categorized as reporting 0, 1, or 2 + life stressors [25].

#### Mental and physical health and unhealthy behaviors

The Study survey includes a variety of validated instruments to screen for mental health conditions. The 17-item Posttraumatic Stress Disorder (PTSD) Checklist − Civilian Version was used to measure the severity of PTSD symptoms [26, 27]. The Patient Health Questionnaire was used to identify depression, panic, other anxiety, and binge eating disorder, respectively [27–29]. Positive screens on these five validated mental health screeners were summed and categorized as 0, 1, or 2 + mental health conditions.

Physical health indicators were identified using a combination of self-reported provider diagnoses, height, and weight. A body mass index greater than 30 kg/m^2^ and four specific diagnoses (hypertension, high cholesterol, migraine, and sleep apnea) were chosen from a list of 38 diagnoses because they were the most prevalent physical health conditions reported on the 2014–2016 survey and were always included on the survey. The sum of the five physical health indicators was categorized as 0, 1, or 2 + .

Unhealthy behaviors included current cigarette smoking, heavy weekly alcohol drinking, and unhealthy sleep duration. Current cigarette smoking was indicated if participants endorsed smoking 100 or more cigarettes in their lifetime and not successfully quitting smoking. Heavy weekly alcohol consumption was determined using the daily number of alcoholic beverages consumed in the past week with a threshold of 7 or 14 drinks per week for females and males, respectively [30]. Unhealthy sleep duration was assessed from a single item coded such that 6 or fewer and 10 or more average hours in a 24-h period indicated unhealthy sleep duration based on National Sleep Foundation recommendations [31]. The sum of the three unhealthy behaviors was categorized as 0, 1, or 2 + .

### Statistical analyses

For each survey wave, we calculated the frequency and percentage of follow-up survey response (yes vs. no) and mode of response (web vs. paper) stratified by panel. Frequencies of military and demographic characteristics, stressful life events, mental and physical health, and unhealthy behaviors were also calculated for eligible participants. In addition, to examine the bivariate relationships between participant characteristics with follow-up survey response, Wave 2 response rate and consistent follow-up response (i.e., responded to all follow-up surveys) were reported for each category of participant-level characteristics. For survey mode, the percentages of web and paper surveys completed were reported among Wave 2 responders and those who responded by one mode more frequently across all follow-up surveys. Multicollinearity was tested among characteristics with a variance inflation factor threshold ≥ 4. Generalized estimating equations (GEEs) were used to estimate the associations between variables of interest and response to follow-up surveys over time. GEEs are a subclass of semiparametric models, which, unlike the parametric generalized linear mixed-effects models, impose no mathematical model for the distributions of the multivariate response due to repeated assessments and thereby provide valid inference for virtually all data distributions [32]. GEEs are not only robust, but also most efficient, or powerful, in the sense that they have the largest power among all such semiparametric models. For both outcomes, survey response and response mode, effect estimates were generated from fully-adjusted models that included all variables of interest [33] (birth year, sex, race and ethnicity, pay grade, service branch, military occupation, LOS, service component, deployment experience, life stressors, mental and physical health, and unhealthy behaviors), panel, and wave were reported. All statistical analyses were conducted using SAS software version 9.4 (SAS Institute, Inc., Cary, North Carolina, USA). The study was approved by the Naval Health Research Center Institution Review Board (protocol number NHRC.2000.0007).

## Results

### Follow-up response rate over time

Eligibility, response rate, and mode of response to surveys are reported in Table 2. Over 93% of participants were eligible (i.e., not deceased, withdrawn from the Study, or completed the 2014-16 short paper survey) at each follow-up wave. Among eligible participants, approximately 60% responded at Wave 2. Furthermore, approximately 70% of the cohort responded to at least one follow-up survey (which may or may not have been Wave 2) and 42% responded to every follow-up survey. However, the follow-up response rate decreased at each consecutive follow-up wave. Among Panels 1–4 participants who completed follow-up surveys between 2006 and 2016, 82% of surveys were completed online.

**Table 2:** Millennium Cohort Study participant eligibility, response, and mode of response between 2001-2003 and 2014-2016 surveys, *n*=201,619

**Panel**	**2001–2003**	**2004–2006**	**2007–2008**	**2011–2013**	**2014–2016**	**No. of surveys completed, by wave** ***n*** **(%)**
**1**	**Enrolled: 77,019 ** Web: 42,167 (55%) Paper: 34,852 (45%)	**Eligible: 76,829 (99%) ** Nonresponder: 21,812 **Responder:** **55,017 (72%)** Web 45,538 (83%) Paper: 9479 (17 %)	**Eligible: 76,044 (99%)** Nonresponder: 21,260 **Responder: ** **54784 (72%)** Web: 46,303 (85%) Paper: 8481 (15%)	**Eligible: 75,419 (98%)** Nonresponder: 23,745 **Responder: ** **51,674 (69%) ** Web: 42,615 (82%) Paper: 9059 (18%)	**Eligible: 71,201 (93%) ** Nonresponder: 23,707 **Responder: ** **47,494 (67%) ** Web: 39,335 (83%) Paper: 8159 (17%)	1: 9336 (12.1) 2: 9154 (11.9) 3: 10,662 (13.8) 4: 12,978 (16.9) 5: 34,889 (45.3)
		Excluded: 190 *	Excluded: 975*	Excluded: 1600 *	Excluded: 5818 *	
**2**		**Enrolled: 31,110 ** Web: 27,250 (88%) Paper: 3860 (12%)	**Eligible: 31,044 (99%) ** Nonresponder: 13,892 **Responder: ** **17,152 (55%)** Web: 14,357 (84%) Paper: 2795 (16%)	**Eligible: 30,842 (99%) ** Nonresponder: 15,693 **Responder: ** **15,149 (49%)** Web: 12,106 (80%) Paper: 3043 (20%)	**Eligible: 29,147 (94%) ** Nonresponder: 15,926 **Responder: ** **13,221 (45%)** Web: 10,968 (83%) Paper: 2253 (17%)	1: 9217 (29.6) 2: 7172 (23.1) 3: 5814 (18.7) 4: 8907 (28.6)
			Excluded: 66*	Excluded: 268*	Excluded: 1963*	
**3**			**Enrolled: 43,438 ** Web: 40,302 (93%) Paper: 3136 (7%)	**Eligible: 43,324 (99%) ** Nonresponder: 21,256 **Responder: ** **22,068 (51%) ** Web: 18,174 (82%) Paper: 3894 (18%)	**Eligible: 41,023 (95%) ** Nonresponder: 23,211 **Responder: ** **17,812 (43%)** Web: 14,943 (84%) Paper: 2869 (16%)	1: 18,061 (41.6) 2: 10,875 (25.0) 3: 14,502 (33.4)
				Excluded: 114*	Excluded: 2415*	
**4**				**Enrolled: 50,052 ** Web: 43,781 (87%) Paper: 6271 (13%)	**Eligible: 47,636 (95%) ** Nonresponder: 22,925 **Responder: ** **24,711 (52%) ** Web: 20,633 (83%) Paper: 4078 (17%)	1: 25,341 (50.6) 2: 24,711 (49.4)
					Excluded: 2416*	

^*^Excluded counts include participants who withdrew from the Study, were deceased at the opening of each survey cycle, or completed the short paper survey at the 2014–2016 cycle

### Descriptive characteristics of follow-up response

Among 198,833 eligible participants, most were male (69%), of non-Hispanic White race and ethnicity (75%), married (58%), senior enlisted personnel (56%), or did not have a college degree (57%). A plurality were born in 1980 or after (42%), served on active duty (42%), or served in the Army (45%) (Table 3).

**Table 3:** Participant characteristics by wave 2 response rate and consistent response rate, *n=*198,833

	***N***	**Wave 2 Responder** **118,948 (59.8)**	**Consistent Responder** **83,011 (41.8)**
		**Row %**	**Row %**
**Panel**			
1	76,829	71.6	45.4
2	31,044	55.3	28.7
3	43,324	50.9	33.5
4	47,636	51.9	51.9
**Sex**			
Male	137,770	59.8	42.5
Female	61,063	59.9	40.1
**Birth year**			
Pre-1960	16,959	81.4	61.9
1960–1969	32,494	76.1	50.2
1970–1979	48,582	62.8	39.1
1980 +	100,798	49.5	36.9
**Race and ethnicity**			
American Indian or Alaskan Native	2821	52.6	36.3
Asian or Pacific Islander	8740	57.7	40.4
Hispanic or Latino	15,708	54.3	36.6
Non-Hispanic Black	24,446	53.7	31.7
Non-Hispanic White	144,670	61.7	44.2
Multiracial	2351	61.8	41.9
Missing	97	69.1	45.4
**Educational attainment**			
High school equivalent or less	42,892	47.3	28.3
Some college, no degree	85,735	55.7	36.5
Associate degree	20,027	65.3	46.7
Bachelor’s degree	34,294	72.7	57.1
Postgraduate degree	15,849	81.2	67.2
Missing	36	50.0	41.7
**Marital status**			
Never married	68,433	54.4	37.0
Married	108,166	63.4	45.1
No longer married	22,226	59.1	39.8
Missing	8	62.5	50.0
**Component status**			
Active duty	71,204	69.4	46.5
Reserve/National Guard	54,599	69.1	48.2
Separated	73,030	43.5	32.3
**Service branch**			
Army	89,011	60.6	41.5
Navy or Coast Guard	35,160	59.6	42.5
Marine Corps	17,130	49.8	35.4
Air Force	57,532	61.8	43.6
**Pay grade**			
Junior enlisted	50,540	45.2	28.5
Senior enlisted	109,936	60.8	41.1
Officer	38,357	76.4	61.1
**Military occupation**			
Admin/supply	61,976	58.8	39.4
Health care	22,383	65.4	48.2
Other	81,519	57.7	40.2
Combat specialist	32,955	63.2	45.5
**Length of service**, years (mean, SD)	10.69 (18.31)	11.62 (17.47)	11.64 (15.39)
**Time deployed**, years (mean, SD)	0.54 (0.70)	0.53 (0.72)	0.55 (0.72)
**Deployment experience**			
Not deployed	128,303	63.5	42.2
Deployed without combat	27,840	52.8	39.8
Deployed with combat	40,196	54.3	42.2
Deployed with unknown combat	2494	37.2	31.6
**Life stressors**			
None	114,112	59.2	42.0
One	50,005	61.9	42.3
More than one	30,178	61.7	41.5
Missing	4538	40.4	31.5
**Mental health**^**a**^			
None	174,765	61.0	42.6
One	12,944	54.3	37.5
More than one	9969	48.9	34.3
Missing	1155	38.4	27.4
**Physical health﻿**^**a**^			
None	136,953	59.1	41.0
One	45,418	61.5	43.4
More than one	15,522	62.4	44.4
Missing	940	40.1	28.9
**Unhealthy behaviors**^**a**^			
None	74,226	63.9	46.3
One	89,619	59.7	41.6
More than one	33,456	52.2	32.9
Missing	1532	37.8	26.9

All characteristics were significantly associated with Wave 2 follow-up survey response and consistent follow-up survey response (*p* < .05)

^a^Mental health indicators included screens for PTSD, depression, panic, other anxiety, and binge eating disorder. Physical health indicators included obese body mass index and diagnoses of hypertension, high cholesterol, migraines, and sleep apnea. Unhealthy behavior indicators included current cigarette smoking, heavy weekly alcohol drinking, and unhealthy sleep duration

Additionally, approximately 60% responded at Wave 2 and 42% responded to all follow-up surveys (Table 3). These rates were at least 5% higher among those born before 1970 compared with those born in 1980 or later, those with a college degree compared with those with a high school equivalent or less, Reserve, National Guard personnel compared with those separated, Officers compared with junior enlisted, health care military occupation compared with administrative or supply. The Wave 2 response rate was also higher among Panel 1 compared with Panel 3 participants and active duty compared with separated personnel, while Panel 4 participants, compared with Panel 2, were more likely to be consistent responders. Wave 2 and consistent responders also had longer length of service, while consistent responders had also deployed for longer.

Finally, among 118,948 Wave 2 responders, 83% completed the web survey and 17% completed the paper survey (Table 4). Similar proportions were observed among the 130,134 participants who responded more often to one survey mode (86% web and 14% paper). Active duty participants, compared with those separated from the military, were more likely to complete web surveys while those separated from the military compared with active duty, junior enlisted compared with Officers, and those who screened positive for more than one mental health condition compared with those who did not screen positive were more likely to complete paper survey (either at Wave 2 or consistently). Those born before 1960 compared with those born between 1960–1969 were more likely to complete paper survey at Wave 2 while Officers, compared with junior enlisted, were more likely to respond using the web survey across all follow-up surveys.

**Table 4:** Participant characteristics by wave 2 mode and preferred follow-up survey mode

	**Wave 2, *n=*118,948**		**Consistent, *n=*130,134**	
	**Web Responder** **98,702 (83.0)**	**Paper Responder** **20,246 (17.0)**	**Web Responder** **111,545 (85.7)**	**Paper Responder** **18,589 (14.3)**
	**Row %**	**Row %**	**Row %**	**Row %**
**Panel**				
1	82.8	17.2	87.1	12.9
2	83.7	16.3	84.5	15.5
3	82.4	17.6	85.6	14.4
4	83.5	16.5	83.5	16.5
**Sex**				
Male	84.0	16.0	86.7	13.3
Female	80.7	19.3	83.3	16.7
**Birth year**				
Pre-1960	77.6	22.4	82.8	17.2
1960–1969	84.7	15.3	88.6	11.4
1970–1979	84.6	15.4	87.6	12.4
1980 +	82.6	17.4	83.8	16.2
**Race and ethnicity**				
American Indian or Alaskan Native	81.4	18.6	83.1	16.9
Asian or Pacific Islander	85.7	14.3	88.1	11.9
Hispanic or Latino	82.8	17.2	85.3	14.7
Non-Hispanic Black	81.7	18.3	83.8	16.2
Non-Hispanic White	83.0	17.0	85.9	14.1
Multiracial	87.0	13.0	90.4	9.6
Missing	76.1	23.9	77.9	22.1
**Educational attainment**				
High school equivalent or less	78.2	21.8	79.6	20.4
Some college, no degree	83.0	17.0	85.2	14.8
Associate degree	83.2	16.8	86.6	13.4
Bachelor’s degree	85.5	14.5	89.4	10.6
Postgraduate degree	85.5	14.5	90.6	9.4
Missing	50.0	50.0	82.6	17.4
**Marital status**				
Never married	81.9	18.1	84.1	15.9
Married	84.0	16.0	87.1	12.9
No longer married	80.7	19.3	83.0	17.0
Missing	80.0	20.0	100.0	0.0
**Component status**				
Active duty	91.2	8.8	93.1	6.9
Reserve/National Guard	83.0	17.0	86.9	13.1
Separated	70.2	29.8	74.2	25.8
**Service branch**				
Army	81.7	18.3	83.7	16.3
Navy or Coast Guard	82.5	17.5	85.8	14.2
Marine Corps	78.3	21.7	81.4	18.6
Air Force	86.3	13.7	89.8	10.2
**Pay grade**				
Junior enlisted	74.0	26.0	75.9	24.1
Senior enlisted	84.4	15.6	87.1	12.9
Officer	86.7	13.3	90.9	9.1
**Military occupation**				
Admin/supply	82.3	17.7	85.0	15.0
Health care	82.7	17.3	85.8	14.2
Other	83.8	16.2	86.4	13.6
Combat specialist	82.4	17.6	85.4	14.6
**Length of service**, years (mean, SD)	11.56 (16.24)	11.92 (22.53)	11.67 (17.35)	11.51 (25.48)
**Time deployed**, years (mean, SD)	0.55 (0.73)	0.45 (0.64)	0.53 (0.71)	0.46 (0.64)
**Deployment experience**				
Not deployed	82.8	17.2	86.1	13.9
Deployed without combat	85.5	14.5	86.7	13.3
Deployed with combat	81.9	18.1	83.5	16.5
Deployed with unknown combat	86.7	13.3	87.4	12.6
**Life stressors**				
None	84.0	16.0	86.8	13.2
One	82.1	17.9	85.0	15.0
More than one	80.5	19.5	83.0	17.0
Missing	86.3	13.7	86.7	13.3
**Mental health**^**a**^				
None	83.6	16.4	86.4	13.6
One	80.0	20.0	81.9	18.1
More than one	74.2	25.8	77.2	22.8
Missing	86.0	14.0	86.3	13.7
**Physical health**^**a**^				
None	83.7	16.3	86.3	13.7
One	81.8	18.2	84.9	15.1
More than one	80.2	19.8	83.0	17.0
Missing	87.0	13.0	88.4	11.6
**Unhealthy behaviors**^**a**^				
None	83.5	16.5	86.8	13.2
One	83.2	16.8	85.9	14.1
More than one	80.6	19.4	82.4	17.6
Missing	86.7	13.3	86.3	13.7

All characteristics were significantly associated with Wave 2 follow-up survey response and consistent follow-up survey response (*p* < .05)

^a^Mental health indicators included screens for PTSD, depression, panic, other anxiety, and binge eating disorder. Physical health indicators included obese body mass index and diagnoses of hypertension, high cholesterol, migraines, and sleep apnea. Unhealthy behavior indicators included current cigarette smoking, heavy weekly alcohol drinking, and unhealthy sleep duration

### Survey response over time

In the adjusted GEE model, all characteristics of interest were significantly associated with follow-up survey response over time except for cumulative years deployed (Table 5). Characteristics positively associated with follow-up survey response over time included being a participant in the initial panel (enrolled between 2001–2003), Wave 3 (compared with Wave 2), increased educational attainment, married, female sex, earlier birth years (i.e., before 1980), and non-Hispanic White race and ethnicity. In addition, serving in the Marine Corps, Navy or Coast Guard (compared with Army), military occupation of combat specialist, active duty (compared with separated participants), junior enlisted pay grade, and deployment experience (with or without combat) were associated with follow-up survey response. Lastly, reporting life stressors, screening positive for mental health conditions, reporting physical health conditions, and reporting no unhealthy behaviors were also associated with follow-up survey response.

**Table 5:** Adjusted GEE effect estimates for follow-up survey response over time

	**Responded**
	**AOR (95% CI)**
**Panel (ref: 1)**	
2	0.70 (0.68, 0.71)
3	0.61 (0.60, 0.63)
4	0.66 (0.64, 0.69)
**Wave (ref: 2)**	
3	1.04 (1.02, 1.05)
4	0.91 (0.90, 0.93)
5	0.85 (0.84, 0.87)
**Female sex (ref: male)**	1.08 (1.06, 1.10)
**Birth year (ref: 1980 +)**	
Pre-1960	3.63 (3.47, 3.79)
1960–1969	2.03 (1.97, 2.10)
1970–1979	1.18 (1.15, 1.21)
**Race and ethnicity (ref: non-Hispanic White)**	
American Indian or Alaskan Native	0.84 (0.78, 0.89)
Asian or Pacific Islander	0.83 (0.80, 0.86)
Hispanic or Latino	0.62 (0.61, 0.64)
Non-Hispanic Black	0.81 (0.78, 0.83)
Multiracial	0.87 (0.80, 0.93)
Missing	0.79 (0.56, 1.10)
**Educational attainment (ref: high school equivalent or less)**	
Some college, no degree	1.37 (1.34, 1.40)
Associate degree	1.87 (1.81, 1.92)
Bachelor’s degree	2.62 (2.54, 2.70)
Postgraduate degree	3.56 (3.41, 3.72)
Missing	0.80 (0.39, 1.65)
**Marital status (ref: never married)**	
Married	1.03 (1.01, 1.05)
No longer married	0.93 (0.90, 0.96)
Missing	0.83 (0.34, 2.05)
**Component status (ref: active duty)**	
Reserve/National Guard	0.78 (0.76, 0.80)
Separated	0.32 (0.31, 0.33)
**Service branch (ref: Army)**	
Navy or Coast Guard	1.04 (1.01, 1.06)
Marine Corps	1.18 (1.14, 1.22)
Air Force	0.93 (0.91, 0.95)
**Pay grade (ref: junior enlisted)**	
Senior enlisted	1.00 (0.98, 1.02)
Officer	0.87 (0.84, 0.90)
**Military occupation (ref: other)**	
Admin/supply	0.90 (0.88, 0.91)
Health care	1.00 (0.98, 1.03)
Combat specialist	1.06 (1.04, 1.09)
**Length of service, 5-year interval**	0.99 (0.99, 0.99)
**Time deployed, 1-year interval**	1.01 (0.99, 1.02)
**Deployment experience (ref: not deployed)**	
Deployed without combat	1.12 (1.09, 1.15)
Deployed with combat	1.21 (1.18, 1.24)
Deployed with unknown combat	0.29 (0.28, 0.30)
**Life stressors (ref: none)**	
One	1.08 (1.06, 1.11)
More than one	1.12 (1.09, 1.15)
Missing	1.08 (0.99, 1.19)
**Mental health**^**a**^ **(ref: none)**	
One	1.15 (1.12, 1.19)
More than one	1.14 (1.10, 1.18)
Missing	0.85 (0.67, 1.08)
**Physical health**^**a**^ **(ref: none)**	
One	1.23 (1.21, 1.25)
More than one	1.58 (1.54, 1.63)
Missing	1.19 (0.99, 1.44)
**Unhealthy behaviors**^**a**^ **(ref: none)**	
One	0.86 (0.84, 0.87)
More than one	0.81 (0.79, 0.83)
Missing	0.87 (0.71, 1.07)

All characteristics were significantly associated with follow-up survey response over time (*p* < .05) except for cumulative years deployed in the adjusted model (*p* = .34)

*AOR* adjusted odds ratio, *CI *confidence interval, *GEE* generalized estimating equation

^a^Mental health indicators included screens for PTSD, depression, panic, other anxiety, and binge eating disorder. Physical health indicators included obese body mass index and diagnoses of hypertension, high cholesterol, migraines, and sleep apnea. Unhealthy behavior indicators included current cigarette smoking, heavy weekly alcohol drinking, and unhealthy sleep duration

### Survey response and mode over time

In the adjusted GEE model, all characteristics were significantly associated with follow-up survey mode over time (Table 6). Certain characteristics, such as increased educational attainment, female sex, earlier birth year, Marine Corps service, deployment experience (with or without combat), screening positive for mental health conditions, and reporting physical health conditions, were associated with higher odds of web and paper survey completion compared with not responding to the follow-up surveys. Other characteristics, such as Wave 3 participation, currently married status, Navy or Coast Guard service, senior enlisted pay grade, and longer time deployed, were only associated with web survey completion. Conversely, those with a military occupation of health care or combat specialist (compared with other), and Reserve/National Guard service (compared with active duty service) were associated only with paper survey completion over time.

**Table 6:** Adjusted GEE effect estimates for follow-up survey response and mode over time

	**Responded:**	
	**Web**	**Paper**
	**AOR (95% CI)**	**AOR (95% CI)**
**Panel (ref: 1)**		
2	0.69 (0.67, 0.71)	0.73 (0.70, 0.76)
3	0.61 (0.59, 0.63)	0.65 (0.62, 0.68)
4	0.66 (0.63, 0.68)	0.68 (0.65, 0.72)
**Wave (ref: 2)**		
3	1.08 (1.06, 1.10)	0.87 (0.85, 0.89)
4	0.96 (0.94, 0.97)	0.76 (0.73, 0.78)
5	0.91 (0.89, 0.93)	0.67 (0.64, 0.69)
**Female sex (ref: male)**	1.04 (1.02, 1.06)	1.29 (1.25, 1.33)
**Birth year (ref: 1980 +)**		
Pre-1960	3.38 (3.21, 3.55)	4.93 (4.65, 5.24)
1960–1969	2.00 (1.93, 2.08)	2.22 (2.11, 2.33)
1970–1979	1.17 (1.14, 1.20)	1.20 (1.16, 1.25)
**Race and ethnicity (ref: non-Hispanic White)**		
American or Alaskan Indian	0.83 (0.77, 0.89)	0.88 (0.79, 0.97)
Asian or Pacific Islander	0.84 (0.80, 0.87)	0.78 (0.74, 0.83)
Hispanic or Latino	0.80 (0.78, 0.83)	0.84 (0.80, 0.87)
Non-Hispanic Black	0.60 (0.59, 0.62)	0.72 (0.70, 0.75)
Multiracial	0.89 (0.82, 0.96)	0.76 (0.68, 0.85)
Missing	0.74 (0.52, 1.06)	0.97 (0.64, 1.48)
**Educational attainment (ref: high school diploma or equivalent or less)**		
Some college, no degree	1.48 (1.45, 1.51)	1.06 (1.03, 1.09)
Associate degree	2.05 (1.99, 2.11)	1.34 (1.28, 1.40)
Bachelor’s degree	2.96 (2.87, 3.06)	1.66 (1.59, 1.74)
Postgraduate degree	4.13 (3.95, 4.32)	1.98 (1.86, 2.10)
Missing	0.64 (0.30, 1.37)	1.54 (0.61, 3.89)
**Marital status (ref: never married)**		
Married	1.05 (1.03, 1.07)	0.94 (0.91, 0.96)
No longer married	0.93 (0.90, 0.96)	0.92 (0.88, 0.96)
Missing	0.84 (0.34, 2.06)	0.89 (0.10, 7.59)
**Component status (ref: active duty)**		
Reserve/National Guard	0.73 (0.71, 0.75)	1.26 (1.21, 1.31)
Separated	0.26 (0.26, 0.27)	0.88 (0.85, 0.91)
**Service branch (ref: Army)**		
Navy or Coast Guard	1.05 (1.03, 1.08)	0.99 (0.95, 1.02)
Marine Corps	1.17 (1.14, 1.22)	1.20 (1.14, 1.25)
Air Force	0.97 (0.95, 0.99)	0.77 (0.75, 0.80)
**Pay grade (ref: junior enlisted)**		
Senior enlisted	1.05 (1.02, 1.08)	0.88 (0.85, 0.91)
Officer	0.92 (0.88, 0.95)	0.72 (0.68, 0.76)
**Military occupation (ref: other)**		
Admin/supply	0.88 (0.86, 0.90)	0.96 (0.93, 0.99)
Health care	0.98 (0.95, 1.01)	1.12 (1.08, 1.17)
Combat specialist	1.02 (0.99, 1.04)	1.24 (1.20, 1.29)
**Length of service, 1-year interval**	0.99 (0.99, 0.99)	0.99 (0.99, 0.99)
**Time deployed, 1-year interval**	1.02 (1.00, 1.03)	0.96 (0.94, 0.98)
**Deployment experience (ref: not deployed)**		
Deployed without combat	1.11 (1.08, 1.14)	1.13 (1.09, 1.18)
Deployed with combat	1.18 (1.15, 1.21)	1.29 (1.25, 1.34)
Deployed with unknown combat	0.25 (0.25, 0.26)	0.47 (0.44, 0.49)
**Life stressors (ref: none)**		
One	1.09 (1.07, 1.11)	1.07 (1.04, 1.10)
More than one	1.12 (1.10, 1.15)	1.09 (1.05, 1.13)
Missing	1.16 (1.05, 1.27)	0.83 (0.72, 0.96)
**Mental health**^**a**^ **(ref: none)**		
One	1.15 (1.11, 1.18)	1.18 (1.14, 1.23)
More than one	1.12 (1.08, 1.16)	1.20 (1.15, 1.26)
Missing	0.82 (0.64, 1.06)	1.00 (0.68, 1.46)
**Physical health**^**a**^ **(ref: none)**		
One	1.25 (1.23, 1.28)	1.14 (1.11, 1.17)
More than one	1.64 (1.60, 1.69)	1.39 (1.34, 1.44)
Missing	1.28 (1.05, 1.56)	0.91 (0.68, 1.21)
**Unhealthy behaviors**^**a**^ **(ref: none)**		
One	0.86 (0.85, 0.88)	0.84 (0.82, 0.86)
More than one	0.81 (0.79, 0.83)	0.81 (0.78, 0.84)
Missing	0.89 (0.72, 1.11)	0.80 (0.56, 1.14)

All characteristics are significantly associated with follow-up web and paper survey response over time (*p* < .05)

*AOR* adjusted odds ratio, *CI* confidence interval, *GEE* generalized estimating equation

^a^Mental health indicators included screens for PTSD, depression, panic, other anxiety, and binge eating disorder. Physical health indicators included obese body mass index and diagnoses of hypertension, high cholesterol, migraines, and sleep apnea. Unhealthy behavior indicators included current cigarette smoking, heavy weekly alcohol drinking, and unhealthy sleep duration

## Discussion

Continued response to follow-up surveys is especially critical for prospective cohort studies, especially for the purpose of ascertaining rare outcomes or those with long latency periods (e.g., cancers, neurodegenerative conditions). It is thus critical to adequately characterize response rates and potential sources of nonresponse bias. Over a follow-up period of 3–15 years, approximately 60% of enrolled participants responded to the first follow-up survey, approximately 70% responded to at least one follow-up survey, and 42% responded at every follow-up survey. Additionally, one notable finding was that Veterans were less likely to respond to follow-up surveys and more likely to complete the paper survey.

To our knowledge, no other longitudinal study of service members and veterans has reported factors associated with follow-up survey response, particularly over multiple waves. We were able to identify military factors associated with follow-up survey response in a longitudinal cohort, such as service in the Navy, Coast Guard or Marine Corps (compared with the Army) and active duty service (compared with Reserve, National Guard, separated from the military). These findings mirror results reported for Panel 1 at Wave 2 [20, 22]. The relatively low response observed among certain groups may be attributable to reasons identified in a previous cross-sectional survey of Air Force personnel: lack of time or interest, attitudes toward sponsoring organization, survey length discouraging initiation or completion of survey, and internet access barriers [34]. Veterans may also feel a diminished connection with a military study.

The observed response rate of 60% in this study was lower than the average response rate of 74% in a recent systematic review and meta-analysis of 141 longitudinal cohort studies [35]. Other longitudinal cohort studies such as the Framingham Study [36], Multi-Ethnic Study of Atherosclerosis [37], Women’s Health Initiative Observational Study [38], and the Nurses’ Health Study [39] have reported higher follow-up response rates (80% or greater). Compared with the populations of interest in other cohort studies, service members are relatively young, highly mobile, extensively surveyed, and have rigorous work schedules and requirements [40] that create challenges for repeated surveying over time. This, combined with differences in timing of follow-up and survey methods and operations, resulted in the lower follow-up survey response rate reported in this analysis. Historically, the Study did not track whether participants were not contacted during survey cycles (e.g., due to undeliverable email addresses or postal addresses that returned mail to sender), so we were unable to distinguish between those not contacted from other nonresponders. Panel 1 Study participants responded at a higher rate (Table 2; average of 70%), which is similar to higher response rates reported in other cohort studies. We conducted a sensitivity analysis with models examining response over time stratified by panel to examine whether there were differences in the associations of the characteristics with response (Supplemental Table), which revealed similar associations across panels. Considering the challenges of retaining enrolled service members and veterans in a longitudinal study, the observed 60% response rate (range 43–71%) is remarkable given the large number of participants that the Study was unable to reach. Even with observed attrition, survey data collected from participants have been extensively analyzed and findings reported in over 150 peer-reviewed publications to date on a wide breadth of topics on the long-term health of service members and veterans [19].

Similar to other cohort studies that have observed declining response rates over time, the Study reported a decline in response rates with each follow-up survey. For example, the Swedish Longitudinal Occupational Survey of Health reported a follow-up response rate of 65% in 2006 that declined to 51% in 2016 [41]. The California Teachers Study reported a response rate of 43% at the sixth survey cycle and found that older participants were more likely to participate than younger participants, which is similar to the results for Panel 1 of the current Study [42]. Demographic characteristics related to survey response among Study participants, such as increased educational attainment, female sex, older age, non-Hispanic White race and ethnicity, have also been reported in other cohort studies [7–9, 43]. The Study has modified its retention methods over time, such as adding endorsement letters, varying the incentives available, and modifying the type and timing of reminders (Table 1). Although beyond the scope of the analyses presented here, it is important for longitudinal cohort studies to adapt their retention strategies and examine the effectiveness of strategies to reduce loss to follow-up. Finally, although survey attributes (e.g., number, order, sensitivity of survey questions) can impact response rates, it is beyond the aims of this paper to summarize the surveys instruments used during the study time-period. A detailed description of the measures and constructs included on the Study's surveys over time can be found elsewhere [44]. Briefly, a core set of questions and constructs remained consistent over time were utilized in these analyses, but the Study was also able to add measures in response to research priorities, military policy, stakeholder input and scientific advances.

Our findings on the associations between life stressors, health status, and unhealthy behaviors on follow-up survey response align with findings from other cohorts. For example, we reported that self-reported physical health conditions were associated with survey response, which is similar to other studies. Baseline responders in Panel 1 were more likely to have certain outpatient diagnoses compared with nonresponders [45]. Studies of other populations also observed higher rates of health care utilization among survey responders [46, 47], which may indicate that survey responders have higher disease burden. In addition, we observed that reporting stressful life experiences was associated with later follow-up response, which was consistent with an analysis that observed higher levels of stress among a population-based post-disaster follow-up questionnaire [48]. Conversely, unhealthy behaviors were associated with lower follow-up survey response, which has been observed in other health surveys [49, 50].

A notable limitation of this analysis is the fact that the Study historically did not track failed contacts (e.g., return to sender, unknown address, or email bounce backs) and we were thus unable to identify and exclude participants who did not receive survey notifications during the survey cycles. Thus, the response rates reported in this analysis may underestimate the true response rates, as the total eligible population would decrease if we were able to exclude those not contacted during follow-up waves. Moving forward, the Study will improve tracking of changes to participant contact information. Additionally, while these analyses allowed for intermittent responses (e.g., it was permissible to respond to Wave 3 even if they failed to respond to Wave 2), analyses did not examine differences in such patterns, which would be a useful avenue for future research. These analyses were not able to examine the impact of participant engagement methods utilized by the Study to increase response, such as incentives, type and frequency of reminders sent over the follow-up period and cannot speak to the influence these have on follow-up survey response. Future work should examine these methodological factors on survey response. Finally, it was beyond the scope of this paper to examine potential nonresponse bias among this cohort. Earlier work among Panel 1 participants observed that nonresponse had minimal impact on study findings [22] and ongoing analyses are updating these results among a contemporary cohort at more recent time points. Despite these limitations, the Study has many strengths, including enrolling a diverse cohort of service members from all service branches, components, pay grades, and occupations; being the longest running military cohort study with follow-up planned through 2068; and having the ability to merge with other data sources to supplement survey data.

## Conclusions

Findings from this analysis indicate that the Study has maintained an adequate response rate over time despite the many challenges associated with surveying military and veteran populations. Additionally, documentation of the different characteristics associated with nonresponse and mode of response is informative for ensuring that appropriate conclusions are drawn over time. These findings can also be instructive for Study strategies for continued participant engagement and may be applicable to other longitudinal cohort studies.

### Supplementary Information

**Additional file 1:** **Supplemental Table.** GEE effect estimates for follow-up survey response over time, stratified by panel.

## Data Availability

The data that support the findings of this study are not currently publicly available because of institutional regulations protecting service member survey responses, but they are available on reasonable request and will require data use agreements to be developed. The data use agreement would need to be approved by the NHRC HIPAA Privacy Officer and ensure that the dataset is truly de-identified based on Safe Harbor and expert determinations. Requests for data access may be sent to the Millennium Cohort Study Principal Investigator: usn.point-loma.navhlthrschcensan.mbx.nhrc-millennium-cohort-pi@health.mil.

## Abbreviations

CTS: Contingency Tracking System
DMDC: Defense Manpower Data Center
GEE: Generalized estimating equation
LOS: Length of service
NHRC: Naval Health Research Center
PTSD: Posttraumatic stress disorder
