# Supplementary material for: Survey response over 15 years of follow-up in the Millennium Cohort Study
Source: BMC Med Res Methodol. 2023 Sep 9;23:205. doi: 10.1186/s12874-023-02018-z (PMC10492282; doi:10.1186/s12874-023-02018-z)
Supplement: Supplementary file 1 — Additional file 1: Supplemental Table. GEE effect estimates for follow-up survey response over time, stratified by panel. [file 12874_2023_2018_MOESM1_ESM.docx]

**Supplemental Table.** GEE Effect Estimates for Follow-up Survey Response Over Time, Stratified by Panel

|  | **All** | | | **Panel 1** | | | **Panel 2** | | | **Panel 3** | | | **Panel 4** | | | |  |
| --- | --- | --- | --- | --- | --- | --- | --- | --- | --- | --- | --- | --- | --- | --- | --- | --- | --- |
|  | **AOR** | **95% CI** | | **AOR** | **95% CI** | | **AOR** | **95% CI** | | **AOR** | **95% CI** | | | **AOR** | **95% CI** | | |
| **Panel (ref: 1)** |  |  |  |  |  |  |  |  |  |  |  |  | |  |  |  | |
| 2 | 0.70 | 0.68 | 0.71 | – | – | – | – | – | – | – | – | – | | – | – | – | |
| 3 | 0.61 | 0.60 | 0.63 | – | – | – | – | – | – | – | – | – | | – | – | – | |
| 4 | 0.66 | 0.64 | 0.69 | – | – | – | – | – | – | – | – | – | | – | – | – | |
| **Wave (ref: 2)** |  |  |  |  |  |  |  |  |  |  |  |  | |  |  |  | |
| 3 | 1.04 | 1.02 | 1.05 | 1.31 | 1.28 | 1.33 | 1.16 | 1.12 | 1.20 | 1.47 | 1.42 | 1.53 | | – | – | – | |
| 4 | 0.91 | 0.90 | 0.93 | 1.11 | 1.08 | 1.14 | 1.02 | 0.98 | 1.07 | – | – | – | | – | – | – | |
| 5 | 0.85 | 0.84 | 0.87 | 1.01 | 0.98 | 1.04 | – | – | – | – | – | – | | – | – | – | |
| **Female sex (ref: male)** | 1.08 | 1.06 | 1.10 | 1.07 | 1.04 | 1.10 | 1.10 | 1.06 | 1.15 | 1.03 | 0.99 | 1.08 | | 1.07 | 1.02 | 1.12 | |
| **Birth year (ref: 1980+)** |  |  |  |  |  |  |  |  |  |  |  |  | |  |  |  | |
| Pre-1960 | 3.63 | 3.47 | 3.79 | 3.49 | 3.28 | 3.71 | 5.28 | 3.78 | 7.38 | 1.47 | 0.79 | 2.73 | | 9.28 | 1.71 | 50.35 | |
| 1960–1969 | 2.03 | 1.97 | 2.10 | 1.96 | 1.86 | 2.07 | 2.25 | 2.03 | 2.50 | 2.01 | 1.67 | 2.43 | | 2.33 | 1.93 | 2.82 | |
| 1970–1979 | 1.18 | 1.15 | 1.21 | 1.16 | 1.11 | 1.22 | 1.09 | 1.04 | 1.14 | 1.20 | 1.14 | 1.27 | | 1.40 | 1.31 | 1.50 | |
| **Race and ethnicity (ref: non-Hispanic White)** |  |  |  |  |  |  |  |  |  |  |  |  | |  |  |  | |
| American Indian or Alaskan Native | 0.84 | 0.78 | 0.89 | 0.83 | 0.74 | 0.94 | 0.87 | 0.74 | 1.02 | 0.78 | 0.69 | 0.88 | | 0.99 | 0.85 | 1.14 | |
| Asian or Pacific Islander | 0.83 | 0.80 | 0.86 | 0.78 | 0.73 | 0.83 | 0.85 | 0.78 | 0.92 | 0.87 | 0.81 | 0.94 | | 0.89 | 0.82 | 0.98 | |
| Non-Hispanic Black | 0.81 | 0.78 | 0.83 | 0.77 | 0.74 | 0.81 | 0.84 | 0.79 | 0.90 | 0.85 | 0.80 | 0.91 | | 0.88 | 0.82 | 0.94 | |
| Hispanic or Latino | 0.62 | 0.61 | 0.64 | 0.62 | 0.60 | 0.64 | 0.67 | 0.63 | 0.71 | 0.66 | 0.62 | 0.70 | | 0.69 | 0.64 | 0.74 | |
| Other | 0.87 | 0.80 | 0.93 | 0.85 | 0.78 | 0.94 | 0.92 | 0.73 | 1.17 | 0.91 | 0.76 | 1.08 | | 0.92 | 0.77 | 1.09 | |
| Missing | 0.79 | 0.56 | 1.10 | 0.74 | 0.48 | 1.14 | 0.76 | 0.44 | 1.32 | – | – | – | | – | – | – | |
| **Educational attainment (ref: high school diploma or equivalent or less)** |  |  |  |  |  |  |  |  |  |  |  |  | |  |  |  | |
| Some college, no degree | 1.37 | 1.34 | 1.40 | 1.29 | 1.24 | 1.33 | 1.50 | 1.44 | 1.57 | 1.50 | 1.44 | 1.57 | | 1.31 | 1.24 | 1.38 | |
| Associate degree | 1.87 | 1.81 | 1.92 | 1.70 | 1.63 | 1.77 | 2.21 | 2.06 | 2.36 | 2.21 | 2.06 | 2.36 | | 1.75 | 1.62 | 1.89 | |
| Bachelor’s degree | 2.62 | 2.54 | 2.70 | 2.26 | 2.16 | 2.37 | 3.45 | 3.21 | 3.72 | 2.96 | 2.74 | 3.19 | | 2.45 | 2.25 | 2.67 | |
| Postgraduate degree | 3.56 | 3.41 | 3.72 | 2.99 | 2.82 | 3.16 | 5.54 | 4.95 | 6.20 | 4.74 | 4.20 | 5.35 | | 3.25 | 2.86 | 3.69 | |
| Missing | 0.80 | 0.39 | 1.65 | 0.44 | 0.11 | 1.84 | 1.60 | 0.71 | 3.63 | 0.52 | 0.08 | 3.24 | | 2.19 | 0.41 | 11.66 | |
| **Marital status (ref: never married)** |  |  |  |  |  |  |  |  |  |  |  |  | |  |  |  | |
| Married | 1.03 | 1.01 | 1.05 | 1.06 | 1.03 | 1.10 | 1.13 | 1.09 | 1.18 | 1.05 | 1.01 | 1.09 | | 0.92 | 0.88 | 0.96 | |
| No longer married | 0.93 | 0.90 | 0.96 | 0.94 | 0.90 | 0.98 | 1.08 | 1.02 | 1.16 | 0.96 | 0.90 | 1.03 | | 0.87 | 0.81 | 0.94 | |
| Missing | 0.83 | 0.34 | 2.05 | 0.91 | 0.44 | 1.88 | 0.92 | 0.14 | 5.86 | – | – | – | | – | – | – | |
| **Component status (ref: active duty)** |  |  |  |  |  |  |  |  |  |  |  |  | |  |  |  | |
| Reserve/National Guard | 0.78 | 0.76 | 0.80 | 0.81 | 0.78 | 0.84 | 0.74 | 0.71 | 0.78 | 0.88 | 0.84 | 0.92 | | 0.70 | 0.66 | 0.74 | |
| Separated | 0.32 | 0.31 | 0.33 | 0.30 | 0.29 | 0.31 | 0.25 | 0.24 | 0.26 | 0.13 | 0.12 | 0.14 | | 0.15 | 0.14 | 0.16 | |
| **Service branch (ref: Army)** |  |  |  |  |  |  |  |  |  |  |  |  | |  |  |  | |
| Navy or Coast Guard | 1.04 | 1.01 | 1.06 | 1.03 | 0.99 | 1.06 | 1.15 | 1.09 | 1.22 | 0.91 | 0.86 | 0.96 | | 1.02 | 0.96 | 1.08 | |
| Marine Corps | 1.18 | 1.14 | 1.22 | 1.06 | 1.00 | 1.12 | 1.43 | 1.33 | 1.54 | 0.91 | 0.86 | 0.96 | | 0.95 | 0.88 | 1.03 | |
| Air Force | 0.93 | 0.91 | 0.95 | 0.93 | 0.90 | 0.96 | 1.05 | 1.00 | 1.10 | 0.83 | 0.79 | 0.87 | | 0.89 | 0.84 | 0.93 | |
| **Pay grade (ref: junior enlisted)** |  |  |  |  |  |  |  |  |  |  |  |  | |  |  |  | |
| Senior enlisted | 1.00 | 0.98 | 1.02 | 1.16 | 1.12 | 1.21 | 1.09 | 1.05 | 1.15 | 1.56 | 1.48 | 1.64 | | 1.21 | 1.14 | 1.28 | |
| Officer | 0.87 | 0.84 | 0.90 | 1.06 | 1.01 | 1.12 | 0.79 | 0.73 | 0.86 | 1.24 | 1.13 | 1.35 | | 1.08 | 0.97 | 1.19 | |
| **Military occupation (ref: other)** |  |  |  |  |  |  |  |  |  |  |  |  | |  |  |  | |
| Admin/supply | 0.90 | 0.88 | 0.91 | 0.88 | 0.86 | 0.91 | 0.96 | 0.92 | 1.00 | 0.91 | 0.87 | 0.95 | | 0.94 | 0.90 | 0.99 | |
| Health care | 1.00 | 0.98 | 1.03 | 0.97 | 0.93 | 1.01 | 1.00 | 0.94 | 1.06 | 1.14 | 1.07 | 1.21 | | 1.14 | 1.07 | 1.23 | |
| Combat specialist | 1.06 | 1.04 | 1.09 | 1.04 | 1.01 | 1.08 | 1.12 | 1.06 | 1.18 | 1.03 | 0.97 | 1.09 | | 1.00 | 0.94 | 1.06 | |
| **Length of service, 5-year interval** | 0.99 | 0.99 | 0.99 | 0.99 | 0.99 | 0.99 | 0.65 | 0.63 | 0.67 | 0.18 | 0.17 | 0.20 | | 0.15 | 0.14 | 0.17 | |
| **Time deployed, years** | 1.01 | 0.99 | 1.02 | 0.99 | 0.97 | 1.01 | 1.06 | 1.03 | 1.09 | 1.09 | 1.06 | 1.12 | | 1.19 | 1.14 | 1.23 | |
| **Deployment experience (ref: not deployed)** |  |  |  |  |  |  |  |  |  |  |  |  | |  |  |  | |
| Deployed without combat | 1.12 | 1.09 | 1.15 | 1.17 | 1.12 | 1.21 | 1.08 | 1.03 | 1.14 | 1.24 | 1.18 | 1.30 | | 1.03 | 0.97 | 1.10 | |
| Deployed with combat | 1.21 | 1.18 | 1.24 | 1.21 | 1.16 | 1.26 | 1.15 | 1.09 | 1.21 | 1.37 | 1.30 | 1.44 | | 1.16 | 1.09 | 1.23 | |
| Deployed with unknown combat | 0.29 | 0.28 | 0.30 | 0.24 | 0.23 | 0.25 | 0.38 | 0.35 | 0.42 | 0.46 | 0.43 | 0.50 | | 1.03 | 0.85 | 1.23 | |
| **Life stressors (ref: none)** |  |  |  |  |  |  |  |  |  |  |  |  | |  |  |  | |
| One | 1.08 | 1.06 | 1.11 | 1.04 | 1.01 | 1.07 | 1.15 | 1.10 | 1.21 | 1.15 | 1.10 | 1.20 | | 1.07 | 1.01 | 1.12 | |
| More than one | 1.12 | 1.09 | 1.14 | 1.08 | 1.05 | 1.12 | 1.12 | 1.06 | 1.19 | 1.19 | 1.12 | 1.27 | | 1.10 | 1.02 | 1.18 | |
| Missing | 1.08 | 0.99 | 1.19 | 0.62 | 0.50 | 0.77 | 0.92 | 0.74 | 1.15 | 0.79 | 0.65 | 0.97 | | 0.62 | 0.52 | 0.73 | |
| **Mental health (ref: none)** |  |  |  |  |  |  |  |  |  |  |  |  | |  |  |  | |
| One | 1.15 | 1.12 | 1.19 | 1.15 | 1.10 | 1.20 | 1.14 | 1.08 | 1.22 | 1.15 | 1.08 | 1.23 | | 1.03 | 0.95 | 1.11 | |
| More than one | 1.14 | 1.10 | 1.18 | 1.12 | 1.06 | 1.17 | 1.14 | 1.06 | 1.21 | 1.11 | 1.03 | 1.20 | | 0.90 | 0.83 | 0.98 | |
| Missing | 0.85 | 0.67 | 1.08 | 0.94 | 0.52 | 1.70 | 0.72 | 0.49 | 1.07 | 1.23 | 0.74 | 2.05 | | 0.83 | 0.55 | 1.24 | |
| **Physical health (ref: none)** |  |  |  |  |  |  |  |  |  |  |  |  | |  |  |  | |
| One | 1.23 | 1.21 | 1.25 | 1.25 | 1.22 | 1.29 | 1.23 | 1.18 | 1.28 | 1.26 | 1.21 | 1.32 | | 1.07 | 1.02 | 1.13 | |
| More than one | 1.58 | 1.54 | 1.63 | 1.64 | 1.59 | 1.70 | 1.55 | 1.45 | 1.66 | 1.52 | 1.42 | 1.63 | | 1.09 | 1.01 | 1.19 | |
| Missing | 1.19 | 0.99 | 1.44 | 1.21 | 0.72 | 2.05 | 1.09 | 0.81 | 1.46 | 0.96 | 0.65 | 1.41 | | 1.48 | 1.03 | 2.13 | |
| **Unhealthy behaviors (ref: none)** |  |  |  |  |  |  |  |  |  |  |  |  | |  |  |  | |
| One | 0.86 | 0.84 | 0.87 | 0.86 | 0.84 | 0.88 | 0.82 | 0.78 | 0.85 | 0.86 | 0.82 | 0.89 | | 0.96 | 0.92 | 1.00 | |
| More than one | 0.81 | 0.79 | 0.83 | 0.83 | 0.80 | 0.86 | 0.79 | 0.75 | 0.83 | 0.79 | 0.75 | 0.83 | | 0.81 | 0.76 | 0.86 | |
| Missing | 0.87 | 0.71 | 1.07 | 0.86 | 0.51 | 1.46 | 0.80 | 0.55 | 1.17 | 0.91 | 0.60 | 1.38 | | 0.92 | 0.68 | 1.24 | |

All variables were statistically significantly associated with response at a *p* < .01, except years deployed (0.47) among Panel 1, sex (0.12), among Panel 3, and mental health (0.05) among Panel 4.
